# Efficacy of acupuncture in managing acute exacerbation of chronic obstructive pulmonary disease: a network meta-analysis

**DOI:** 10.3389/fmed.2026.1786971

**Published:** 2026-04-21

**Authors:** Haogeng Wang, Fan Zhang, Yiming Lin, Xiaona Ye, Yujie Li, Yuxia Ma

**Affiliations:** 1 School of Acupuncture and Tuina, Shandong University of Traditional Chinese Medicine, Jinan, China; 2 First Clinical Medical College, Shandong University of Traditional Chinese Medicine, Jinan, China; 3 The Second Affiliated Hospital of Shandong University of Traditional Chinese Medicine, Jinan, China; 4 Key Laboratory of Traditional Chinese Medicine Classical Theory, Ministry of Education, Shandong University of Traditional Chinese Medicine, Jinan, China

**Keywords:** acupuncture, chronic obstructive pulmonary disease, FEV1, FVC, network meta-analysis

## Abstract

**Background:**

This study aimed to evaluate the efficacy of acupuncture-related interventions for acute exacerbation of chronic obstructive pulmonary disease (AECOPD) using a network meta-analysis.

**Methods:**

PubMed, Embase, Cochrane Library, Web of Science, CNKI, VIP, and Wan fang were searched from inception to September 20, 2025. Randomized controlled trials involving patients with AECOPD and evaluating acupuncture-related interventions, alone or in combination with usual treatment, were included. The primary outcome was symptom improvement. Secondary outcomes were forced expiratory volume in 1 s (FEV1), forced vital capacity (FVC), and FEV1/FVC. A Bayesian network meta-analysis was conducted to synthesize direct and indirect evidence across interventions.

**Results:**

A total of 45 randomized controlled trials involving 3,156 participants were included. Compared with usual treatment, several acupuncture-related interventions were associated with improvements in symptom-related outcomes and pulmonary function parameters. For symptom improvement, acupoint application (AA) and abdominal needle (AN) showed statistically significant associations compared with usual treatment (AA: OR = 4.97, 95% CrI 3.18–8.24; AN: OR = 6.84, 95% CrI 2.59–21.54). For pulmonary function outcomes, thunder-fire moxibustion (TFM) was associated with higher FEV1 values (MD = 0.71, 95% CrI 0.50–0.92), while conventional acupuncture (AC) and ear acupuncture (EAC) were associated with improvements in FVC and FEV1/FVC. Ranking analyses suggested that AN, TFM, AC, and AA/EAC tended to rank relatively higher for symptom improvement, FEV1, FVC, and FEV1/FVC, respectively; however, surface under the cumulative ranking curve (SUCRA) values reflect ranking probabilities rather than the magnitude of treatment effects.

**Conclusion:**

Acupuncture-related interventions may provide potential adjunctive benefits for patients with AECOPD when used in combination with conventional treatment. In this network meta-analysis, several interventions were associated with improvements in symptom-related outcomes and pulmonary function parameters. However, ranking results from SUCRA should be interpreted cautiously and considered together with effect estimates and the certainty of evidence. Given that most included trials evaluated acupuncture as an adjunct to usual care and the overall certainty of evidence ranged from moderate to low, further large-scale and well-designed randomized controlled trials are needed to confirm these findings.

**Systematic review registration:**

https://www.crd.york.ac.uk/PROSPERO/view/CRD420251080558, identifier CRD420251080558.

## Background

Chronic obstructive pulmonary disease (COPD) is a common chronic respiratory disorder characterized by long-term airway inflammation, airflow limitation, and declining lung function. COPD has become a major cause of mortality and disability worldwide, particularly among the elderly (1, 2). According to the World Health Organization (WHO), COPD ranks as the third leading cause of death globally, with projections indicating it will continue to rise by 2030 (3). Among the different clinical phases of COPD, acute exacerbations of chronic obstructive pulmonary disease (AECOPD) represent a critical and unstable stage characterized by an acute worsening of respiratory symptoms beyond normal day-to-day variation. AECOPD is typically accompanied by intensified airway inflammation, increased mucus secretion, aggravated airflow limitation, and impaired gas exchange. These episodes often require urgent medical intervention or hospitalization and are strongly associated with accelerated lung function decline, higher mortality, and substantial healthcare burden (4, 5). Unlike stable COPD, which primarily focuses on long-term symptom control and functional maintenance, AECOPD requires rapid control of acute inflammation and bronchoconstriction to prevent respiratory deterioration.

Currently, AECOPD treatment relies heavily on pharmacological interventions, particularly antibiotics, corticosteroids, and bronchodilators (6). While these therapies can alleviate symptoms and improve lung function, many patients still face incomplete symptom improvement, frequent relapses, and persistent challenges in long-term disease control (7). Moreover, pharmacological treatments carry potential side effects, such as osteoporosis and immunosuppression from prolonged corticosteroid use. Given the acute inflammatory burden and clinical urgency of AECOPD, there remains a need for adjunctive therapies that can enhance symptom control while minimizing treatment-related side effects (8).

Acupuncture, as one of the traditional Chinese medicine therapies, has gained increasing attention in COPD treatment in recent years (9). By stimulating specific acupoints to regulate qi and blood flow and unblock meridians, acupuncture has demonstrated therapeutic effects for various conditions. In COPD treatment, acupuncture alleviates dyspnea and related symptoms through mechanisms including promoting airway dilation, reducing inflammatory responses, and improving pulmonary blood circulation (10, 11). A growing body of clinical studies and small-scale trials suggests acupuncture holds potential for relieving COPD symptoms, enhancing lung function, and improving patients’ quality of life. Recent clinical studies increasingly demonstrate acupuncture’s efficacy in improving COPD symptoms, enhancing lung function, and reducing the frequency of acute exacerbations (12). Numerous investigations reveal that acupuncture significantly alleviates dyspnea, coughing, and sputum production in COPD patients while positively impacting their quality of life. Some studies also indicate that acupuncture, as a non-pharmacological treatment, when combined with conventional drug therapy, may help reduce medication dosage, minimize side effects, and achieve better therapeutic outcomes (13, 14).

However, it is important to note that most existing studies focus on patients with stable COPD rather than those experiencing acute exacerbations. The pathophysiological mechanisms and therapeutic goals of AECOPD differ substantially from stable disease, and evidence specific to AECOPD remains fragmented and inconclusive. Therefore, a focused evaluation of acupuncture specifically in the context of AECOPD is warranted. Traditional pairwise meta-analysis is limited to synthesizing evidence from head-to-head comparisons and cannot simultaneously compare multiple competing interventions across a connected evidence network. Consequently, it is unable to determine the relative comparative effectiveness among various acupuncture modalities or to establish a hierarchy of treatment symptom improvement.

Network meta-analysis (NMA) allows the integration of both direct and indirect evidence within a unified analytical framework, enabling simultaneous comparison of multiple interventions while preserving randomization. Under the assumptions of transitivity and consistency, NMA provides more comprehensive comparative estimates and permits probabilistic ranking of interventions according to efficacy.

Given the diversity of acupuncture-related interventions and the absence of direct head-to-head comparisons among them, conducting an NMA offers incremental methodological and clinical value by identifying the most potentially effective acupuncture strategy for AECOPD management.

## Methods

This systematic evaluation and meta-analysis would strictly follow the PRISMA (Preferred Reporting Items for Systematic Reviews and Meta-Analyses) guidelines (15). And it is registered in Prospero with registration number CRD420251080558.

### Literature retrieval

This study conducted a systematic literature search across multiple medical databases, including PubMed, Embase, Cochrane Library, Web of science, CNKI, VIP, and Wan fang. We employed keyword combinations including “COPD,” “acute exacerbation,” “acupuncture,” and “randomized controlled trial” to ensure coverage of all relevant high-quality randomized controlled trials (RCTs). The search period spanned from the inception of each database to September 20, 2025. The specific search strategy is detailed in Supplementary Table S1.

### Inclusion and exclusion criteria

The studies included in this review must meet the following criteria: Only randomized controlled trials (RCTs) involving patients diagnosed with COPD an acute exacerbation according to recognized (COPD diagnosis must be established according to recognized international or national guidelines (the Global Initiative for Chronic Obstructive Lung Disease criteria), defined by a post-bronchodilator FEV1/FVC < 0.70. AECOPD was operationally defined as an acute worsening of respiratory symptoms (increased dyspnea, cough, and/or sputum production) beyond normal day-to-day variation, requiring additional treatment (systemic corticosteroids, antibiotics, hospitalization, or escalation of respiratory support) were included. The studies must have involved acupuncture treatment and could be compared with Usual treatment (UT) (pharmacotherapy, oxygen therapy, respiratory training). Primary outcome measures focused on symptom improvement, Secondary outcomes: FEV1, FVC, and the FEV1/FVC. No language restrictions were applied, allowing inclusion of both Chinese and English studies.

Exclusion criteria included: non-randomized controlled trials (observational studies, case-control studies, cohort studies); studies involving only stable-phase COPD or lacking acute exacerbation symptoms; studies not involving acupuncture treatment or acupuncture combined with other therapies; studies failing to report efficacy or pulmonary function data; all animal studies, *in vitro* research, and other non-clinical studies; and studies with incomplete data or data unsuitable for meta-analysis.

### Definition of interventions and network nodes

To improve transparency and interpretability of the treatment network, interventions were categorized into predefined nodes according to the actual treatment strategies reported in the included trials. These nodes included usual treatment (UT), acupoint application (AA), abdominal needle (AN), conventional acupuncture (AC), ear acupuncture (EAC), press acupuncture (PA), thunder-fire moxibustion (TFM), warm acupuncture (WA), transcutaneous electrical nerve stimulation (TENS), acupoint application combined with traditional Chinese medicine (AA_TCM), and acupoint application combined with ear point embedding (AA_EPE), where applicable.

UT referred to conventional medical management for AECOPD as reported in individual trials, which could include pharmacotherapy, oxygen therapy, respiratory support, and other supportive care. Because some nodes represented combination strategies rather than isolated acupuncture modalities, the corresponding comparisons were interpreted as comparisons between treatment packages rather than the independent effect of acupuncture alone.

### Data extractions

Two authors independently screened the literature for inclusion by importing the literature into endnote according to the literature inclusion and exclusion criteria, the final included studies were used for data extraction using excel software and if there was a dispute about the literature screening then it would be discussed, or a third person would be sought to adjudicate. The extracted data contained basic characteristics of the study (first author, year of publication), basic characteristics of the population (sample size, gender, age), intervention, and outcome.

### Risk of bias

In the Meta-analysis of this study, we used the ROB 2.0 (Risk of Bias 2.0) tool (16) to assess the risk of bias of the included studies. Developed by the Cochrane Collaboration, the ROB 2.0 tool is a standardized tool for assessing the risk of bias in RCTs, which is aimed at systematically identifying and evaluating bias factors that may affect the validity of the results of the study. This improves the accuracy and reliability of Meta-analyses. The ROB 2.0 tool contains five key assessment domains: randomization process, intervention implementation, outcome measures, data reporting, and other sources of bias. Each domain is scored according to the transparency, reasonableness, and potential for bias in the study design and implementation, and is categorized as low risk, high risk, and uncertain risk. In conducting the assessment, two independent reviewers will score each domain based on the specifics of the study.

### Grade assessment

To evaluate the certainty of evidence in this network meta-analysis, we applied the GRADE (17) (Grading of Recommendations Assessment, Development and Evaluation) approach adapted for network meta-analysis. In addition to the conventional five GRADE domains (risk of bias, inconsistency, indirectness, imprecision, and publication bias), we incorporated NMA-specific considerations. The transitivity assumption was examined by assessing the similarity of included trials with respect to clinical and methodological characteristics (e.g., patient severity, intervention type, comparator, and outcome definitions). Network inconsistency (incoherence) between direct and indirect evidence was evaluated using statistical inconsistency models and node-splitting analysis. Between-study heterogeneity was assessed using I^2^ and τ^2^ statistics within the network framework. We also considered the relative contribution of direct and indirect evidence to each network estimate when rating certainty. The overall certainty of evidence for each comparison was categorized as high, moderate, low, or very low, in accordance with GRADE guidance for network meta-analysis and principles outlined in the CINeMA framework.

### Statistical analysis

In this study, statistical analysis will be performed using a Bayesian framework for Network Meta-Analysis (NMA) (18) to compare the symptom improvement of different acupuncture in AECOPD. The primary outcome indicators include symptom improvement, FEV1, FVC, and the FEV1/FVC. First, a treatment network will be constructed by connecting studies that directly or indirectly compare two or more treatments. Each treatment will serve as a node, and edges between nodes represent direct comparisons between treatments performed in individual studies. Next, Bayesian network Meta-analysis was employed, using mean difference (MD) or odd ratio (OR) as an indicator of treatment effect for continuous or dichotomous variables, a Bayesian approach allowing the introduction of prior distributions and the use of Markov chain Monte Carlo (MCMC) methods to generate posterior distributions of treatment effects. In this study, Deviance Information Criterion (DIC) will be used to assess the consistency of the network Meta-analysis model. Specifically, two models will be constructed: one assuming consistency and the other assuming inconsistency. By comparing the DIC values of these two models, we can determine whether there is inconsistency in the network. To account for inter-study heterogeneity, a random effects model will be used for the analysis. Heterogeneity will be assessed by the I^2^ statistic, with significant heterogeneity indicated if the I^2^ value exceeds 50%. Network consistency will also be tested to ensure consistency between direct and indirect evidence. If inconsistency is found, possible causes will be explored, and the model will be adjusted through sensitivity analysis. All statistical analyses will be performed using R software (version 4.0.0) and the “gemtc” package, which is specifically designed for Bayesian network meta-analysis. The results will be presented in the form of posterior mean estimates, confidence intervals (95% CrI) and ranking probabilities, thus providing reliable conclusions on the comparison of treatments.

## Results

### Literature retrieval results

**FIGURE 1:**
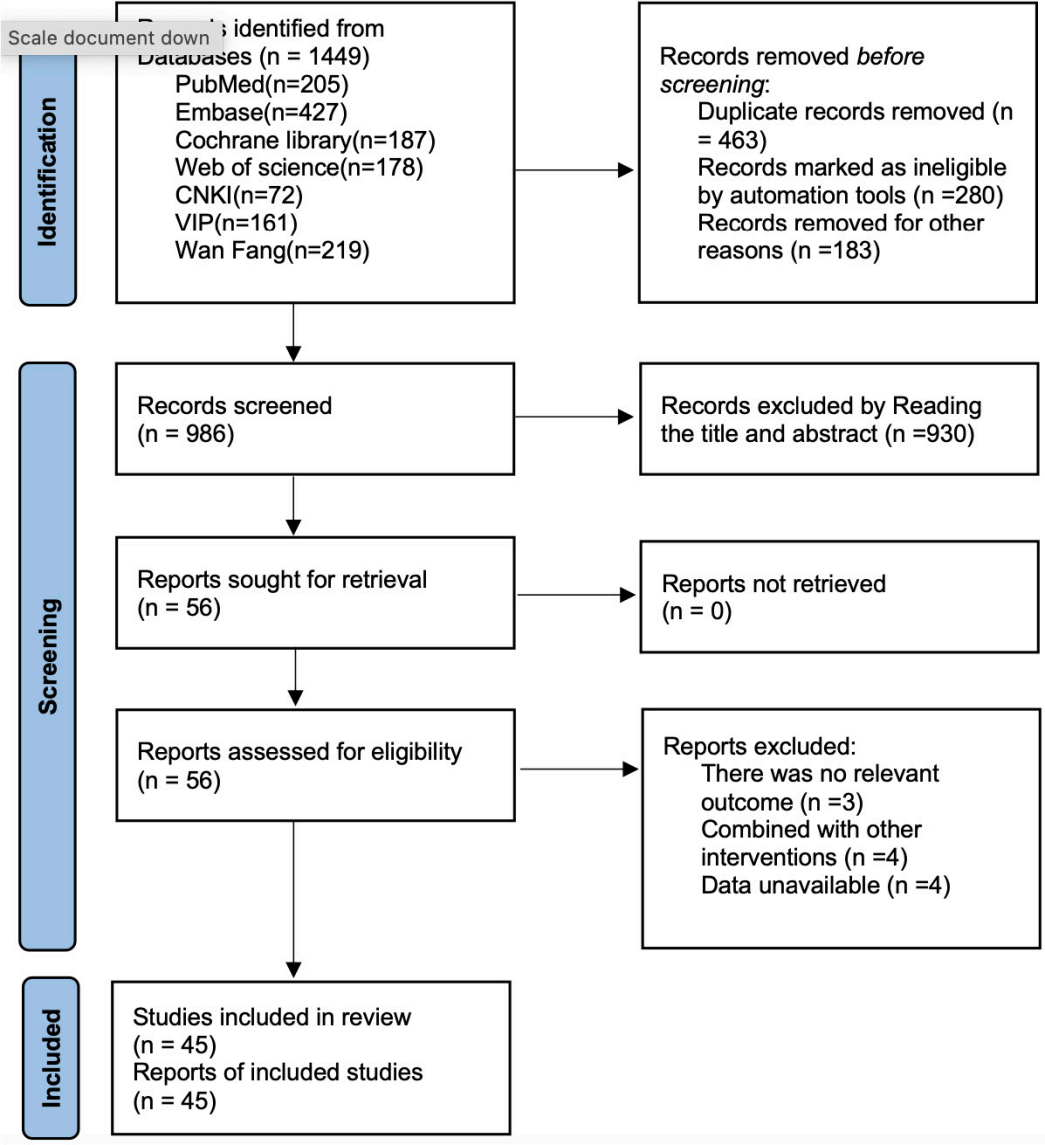
Literature search flow chart.

This study retrieved a total of 1,449 articles through searches of PubMed (*n* = 205), Embase (*n* = 427), Cochrane Library (*n* = 187), Web of Science (*n* = 178), CNKI (*n* = 72), VIP (*n* = 161), and Wan Fang (*n* = 219), a total of 1,449 articles were identified. After removing 463 duplicate records, 930 articles were excluded based on title and abstract screening, and 11 full-text articles were excluded (There was no relevant outcome (*n* = 3), Combined with other interventions (*n* = 4), Data unavailable (*n* = 4)). Ultimately, 45 articles (10, 19–62) were included. The search flowchart is shown in Figure 1.

### Basic characteristics of included study

A total of 45 RCTs involving 3,156 participants were included. The treatment network comprised the following predefined nodes: UT, AA, AN, AC, EAC, PA, TFM, WA, TENS, AA_TCM, and AA_EPE. The male/female ratio was 1,663/1,493. Detailed baseline characteristics are presented in Table 1.

**TABLE 1:** Table of basic characteristics.

Study	Year	Sample size	Gender (M/F)	Mean age	Intervention	Outcomes	Efficacy defined
Oncu	2017	TENS:35 UT:35	54/16	TENS:65 UT:65	TENS:45 min/days	FEV1; FVC	
CJ Shangguan	2021	AA_TCM:35 UT:35	47/23	AA_TCM:76.02 UT:75.67	AA: 2 weeks; Feishu (BL 23), Shenshu (BL 23), Pishu (BL 23), Fengmen (BL 7), Guanyuan (CV 4) and Tiantu (BL 7)	Efficacy; FEV1; FVC; FEV1/FVC	Relief of clinical symptoms
W Yan	2024	AC:50 UT:50	61/39	AC:62.01 UT:61.91	AC: Yinjiao, Tianshu, Danzhong, Taiyuan, Taixi, Shuangpishu, Shuangfeishu acupoints	Efficacy; FEV1; FVC	Relief of clinical symptoms
L Yu	2019	AA_TCM:42 UT:42	45/39	AA_TCM:66.51 UT:67.42	AA: Feishu points, Dachangshu points, Tiantu points, Pishu points, Shenshu points	Efficacy; FEV1; FVC; FEV1/FVC	Relief of clinical symptoms
Y He	2024	AA:50 UT:50	NR	NR	AA: Feishu points, Dachangshu points, Tiantu points, Pishu points	Efficacy	Relief of clinical symptoms
F He	2019	AC:60 UT:60	69/51	AC:66.5 UT:66.2	AC: Feishu, Pishu, Shenshu, Danzhong, Qihai, Dazhui, Dingchuan, Zusanli, Guanyuan, Yinlingquan, Quchi points	Efficacy; FEV1; FVC	Relief of clinical symptoms
W Guan	2016	AN:34 UT:34	49/18	AN:65.17 UT:66.53	AN: Zhongwan, Xiawan, Qihai, Guanyuan, Huaroumen, Tianshu, Daheng, Dai Mai	Efficacy; 6MWT	Relief of clinical symptoms
YZ Zhuo	2019	AA_EPE:53 UT:53	58/48	AA_EPE:53.5 UT:52.7	AA: Feishu points, Dachangshu points, Tiantu points, Pishu points	Efficacy; FEV1; FVC; FEV1/FVC	Relief of clinical symptoms
JY Lu	2021	AA:60 UT:60	76/44	AA:60.79 UT:61.24	AA: Feishu points, Dachangshu points, Tiantu points, Pishu points	Efficacy; FEV1/FVC	Relief of clinical symptoms
JY Lu	2022	AA:42 UT:42	58/26	AA:63 UT:64	AA: Feishu points, Dachangshu points, Tiantu points, Pishu points	Efficacy; FEV1; FVC; FEV1/FVC	Relief of clinical symptoms
ZD Xiang	2021	AA:53 UT:53	73/33	AA:64.98 UT:64.67	AA: Feishu points, Dachangshu points, Tiantu points, Pishu points	Efficacy; FEV1; FEV1/FVC	Relief of clinical symptoms
MJ Wu	2025	AC:33 UT:33	36/30	AC:65.1 UT:64.78	AC: Feishu, Pishu, Shenshu, Danzhong, Qihai, Dazhui, Dingchuan, Zusanli, Guanyuan, Yinlingquan, Quchi points	Efficacy; 6MWT	Relief of clinical symptoms
SM Zhou	2023	AA:30 UT:30	48/12	AA:72.37 UT:72.85	AA: Feishu points, Dachangshu points, Tiantu points, Pishu points	Efficacy; FEV1; FVC; FEV1/FVC	Relief of clinical symptoms
WD Zhou	2025	AA_TCM:31 UT:31	39/23	AA_TCM:63.94 UT:64.39	AA: Feishu points, Dachangshu points, Tiantu points, Pishu points, Shenshu points	FEV1/FVC; 6MWT	
QH Xia	2021	AA_TCM:38 UT:38	61/15	AA_TCM:72.14 UT:73.16	AA: Feishu points, Dachangshu points, Tiantu points, Pishu points, Shenshu points	Efficacy; FEV1/FVC	Relief of clinical symptoms
L Jiang	2024	AA:30 UT:30	33/27	AA:62.43 UT:62.35	AA: Feishu points, Dachangshu points, Tiantu points, Pishu points, Shenshu points	Efficacy; FEV1; FVC	Relief of clinical symptoms
J Meng	2025	AA_TCM:40 UT:40	42/38	AA_TCM:65.54 UT:65.72	AA: Feishu points, Dachangshu points, Tiantu points, Pishu points, Shenshu points	FEV1; FVC; FEV1/FVC	
WJ Song	2023	AA_TCM:55 UT:55	59/51	AA_TCM:64.5 UT:64.21	AA: Feishu points, Dachangshu points, Tiantu points, Pishu points, Shenshu points	FEV1; FVC; FEV1/FVC	
DF Shi	2025	EAC: 49 UT:49	52/46	EAC: 71.7 UT:71.8	EAC: Pulmonary point, tracheal point and subcortical	Efficacy; FEV1; FVC; FEV1/FVC	Relief of clinical symptoms
T Liao	2015	AA:40 UT:40	48/32	AA:70.2 UT:69.3	AA: Feishu points, Dachangshu points, Tiantu points, Pishu points	Efficacy; FEV1; FEV1/FVC	Relief of clinical symptoms
Q Zhang	2022	AC:35 UT:35	33/37	AC:57.63 UT:56.55	AC: Yinjiao, Tianshu, Danzhong, Taiyuan, Taixi, Shuangpishu, Shuangfeishu acupoints	Efficacy; FEV1; FVC; FEV1/FVC	Relief of clinical symptoms
HP Xu	2021	AA_TCM:40 UT:40	55/35	AA_TCM:76.73 UT:77.62	AA: Feishu points, Dachangshu points, Tiantu points, Pishu points, Shenshu points	FEV1; FVC;	
YJ Yi	2025	AA:30 UT:30	37/23	AA:63.85 UT:63.89	AA: Feishu points, Dachangshu points, Tiantu points, Pishu points, Shenshu points	Efficacy; FEV1; FVC; FEV1/FVC	Relief of clinical symptoms
X Jing	2017	AA_TCM:45 UT:45	52/38	AA_TCM:66.43 UT:65.81	AA: Feishu points, Dachangshu points, Tiantu points, Pishu points, Shenshu points	Efficacy	Relief of clinical symptoms
PJ Zhu	2024	PA:40 UT:40	51/29	PA:62.81 UT:63.25	PA: Chize, Dingchuan, Feishu, Hegu, Shanzhong, Lieque, Zusanli	Efficacy; FEV1; FVC; FEV1/FVC	Relief of clinical symptoms
Y Li	2024	AA_TCM:40 UT:40	50/30	AA_TCM:70.3 UT:70.28	AA: Feishu points, Dachangshu points, Tiantu points, Pishu points, Shenshu points	Efficacy; FVC; FEV1/FVC	Relief of clinical symptoms
AC Li	2020	AA:54 UT:54	67/41	AA:65.23 UT:64.69	AA: Feishu points, Dachangshu points, Tiantu points, Pishu points, Shenshu points	FEV1	
C Li	2024	AA_TCM:50 UT:50	55/45	AA_TCM:71.26 UT:70.46	AA: Feishu points, Dachangshu points, Tiantu points, Pishu points, Shenshu points	Efficacy; FEV1/FVC	Relief of clinical symptoms
DL Yang	2024	AC:45 UT:45	54/36	AC:58.36 UT:60.11	AA: Feishu points, Dachangshu points, Tiantu points, Pishu points, Shenshu points	FEV1; FVC;	
FX Liang	2020	AA:40 UT:40	62/18	AA:64.32 UT:64.27	AA: Feishu points, Dachangshu points, Tiantu points, Pishu points, Shenshu points	Efficacy; FEV1; FVC;	Relief of clinical symptoms
X Ou	2022	AA:55 UT:54	59/50	AA:65.45 UT:66.34	AA: Feishu points, Dachangshu points, Tiantu points, Pishu points	Efficacy; FEV1; FVC; FEV1/FVC	Relief of clinical symptoms
JY Wang	2015	AC:32 UT:31	35/28	64	AC: Yinjiao, Tianshu, Danzhong, Taiyuan, Taixi, Shuangpishu, Shuangfeishu acupoints	Efficacy; FVC;	Relief of clinical symptoms
AJ Shen	2025	AA:45 UT:47	50/42	18–75	AA: Feishu points, Dachangshu points, Tiantu points, Pishu points, Shenshu points	FEV1/FVC	
JW Wen	2024	AC:43 UT:43	46/40	AC:72.09 UT:72.12	AC: Yinjiao, Tianshu, Danzhong, Taiyuan, Taixi, Shuangpishu, Shuangfeishu acupoints	Efficacy; FVC; FEV1/FVC	Relief of clinical symptoms
XD Pan	2024	TFM:35 UT:32	36/29	TFM:71 UT:69	TFM: Feishu, Pishu, Shenshu, Dingchuan, Danzhong, Shenque, Zusanli and Fenglong points	Efficacy; FEV1; FEV1/FVC	Relief of clinical symptoms
XD Pan	2025	AC:40 UT:36	40/36	AC:63.25 UT:63.31	AC: Yinjiao, Tianshu, Danzhong, Taiyuan, Taixi, Shuangpishu, Shuangfeishu acupoints	Efficacy; FEV1; FVC; FEV1/FVC	Relief of clinical symptoms
Q Wang	2024	EAC:45 UT:45	50/40	EAC:61.53 UT:62.87	EAC: Pulmonary point, tracheal point and subcortical	Efficacy; FEV1/FVC	Relief of clinical symptoms
XC Wang	2022	AA_EPE:46 UT:46	54/38	AA_EPE:62.34 UT:63.03	AA: Feishu points, Dachangshu points, Tiantu points, Pishu points	FEV1; FVC; FEV1/FVC	
YX Wang	2022	AA:43 UT:43	50/36	AA:75.65 UT:73.69	AA: Feishu points, Dachangshu points, Tiantu points, Pishu points	FEV1; FVC; FEV1/FVC	
Y Wang	2020	AN:50 UT:50	47/53	AN:62.17 UT:64.44	AN: Zhongwan, Xiawan, Qihai, Guanyuan, Huaroumen, Tianshu, Daheng, Dai Mai	Efficacy	Relief of clinical symptoms
F Xie	2019	WA:30 UT:30	26/34	WA:64 UT:62	WA: Fenglong, Feishu, Taiyuan, Zusanli, Zhongfu, Yinlingquan	FEV1; FVC; FEV1/FVC	
X Xie	2023	PA:39 UT:39	NR	40–80	PA: Chize, Dingchuan, Feishu, Hegu, Shanzhong, Lieque, Zusanli	Efficacy; FEV1; FEV1/FVC	Relief of clinical symptoms
X Zhao	2019	AA:25 UT:25	26/24	AA:60.8 UT:61.1	AA: Feishu points, Dachangshu points, Tiantu points, Pishu points	Efficacy; FEV1/FVC	Relief of clinical symptoms
B Chen	2023	AC:42 UT:42	NR	NR	AC: Yinjiao, Tianshu, Danzhong, Taiyuan, Taixi, Shuangpishu, Shuangfeishu acupoints	Efficacy; FVC; FEV1/FVC	Relief of clinical symptoms
Y Long	2024	AC:30 UT:30	33/27	AC:62.36 UT:62.79	AC: Yinjiao, Tianshu, Danzhong, Taiyuan, Taixi, Shuangpishu, Shuangfeishu acupoints	Efficacy; FEV1; FVC; FEV1/FVC	Relief of clinical symptoms

TENS, transcutaneous electrical nerve stimulation; AA, acupoint application; TCM, Traditional Chinese medicine; AN, Abdominal needle; EPE, Ear Point Embedding; EAC, Ear acupuncture; PA, Press acupuncture; MOX, Moxibustion; TFM, Thunder fire moxibustion; WA, Warm acupuncture; NR, not reported; FEV1, Forced Expiratory Volume in 1 second; FVC, Forced Vital Capacity; UT, Usual treatment.

### Risk of bias results

The risk-of-bias assessment is presented in Supplementary Figures S1, S2. Most studies reported random allocation, although the details of sequence generation and allocation concealment were not always sufficiently described. For the domain of deviations from intended interventions, many studies did not clearly report blinding of participants or personnel, which is a common challenge in acupuncture trials. Therefore, judgments in this domain were based on the likelihood that lack of blinding could have influenced the outcomes and whether co-interventions appeared balanced across groups. Overall, several studies were judged as having some concerns because of limited reporting, while relatively few studies provided sufficient methodological detail to support low-risk judgments across all domains.

The certainty of evidence was rated as moderate for symptom-related outcomes and low for pulmonary function outcomes, mainly because of concerns regarding risk of bias, imprecision, and inconsistency. Detailed downgrading decisions are provided in Supplementary Table S2.

### Pairwise meta-analysis

This study employed a pairwise meta-analysis. Results (Table 2) indicate that for symptom improvement, UT vs. AA [OR = 0.20, 95% CrI (0.12, 0.32)], UT vs. AA_TCM [OR = 0.24, 95% CrI (0.13, 0.41)], UT vs. AC [OR = 0.25, 95% CrI (0.15, 0.40)], UT vs. AN [OR = 0.15, 95% CrI (0.05, 0.39)]. For FEV1, UT vs. AA [MD = −0.33, 95% CrI (−0.56, −0.10)], UT vs. AA_EPE [MD = −0.49, 95% CrI (−1.00, −0.02)], UT vs. AA_TCM [MD = −0.35, 95% CrI (−0.68, −0.03)], UT vs. AC [MD = −0.43, 95% CrI (−0.73, −0.14)]. For FVC, UT vs. AA [MD = −0.31, 95% CrI (−0.61, −0.19)], UT vs. AA_TCM [MD = −0.39, 95% CrI (−0.72, −0.06)], UT vs. AC [MD = −0.58, 95% CrI (−0.86, −0.30)]. For FEV1/FVC, UT vs. AA [MD = −5.07, 95% CrI (−7.55, −1.63)], UT vs. AA_EPE [MD = −5.07, 95% CrI (−9.65, −0.50)], UT vs. AA_TCM [MD = −5.60, 95% CrI (−8.09, −3.27)], UT vs. EAC [MD = −9.38, 95% CrI (−14.20, −4.63)].

**TABLE 2:** Results of Pairwise meta-analysis.

Outcomes	Pairwise meta-analysis	No of study	Heterogeneity (%)	OR 95%CrI
Efficacy	UT vs. AA	11	0	0.20 (0.12, 0.32)
	UT vs AA_EPE	1	NA	0.26(0.05, 1.08)
	UT vs. AA_TCM	6	0	0.24(0.13, 0.41)
	UT vs. AC	9	33.9	0.25(0.15, 0.40)
	UT vs. AN	2	0	0.15(0.05, 0.39)
	UT vs. EAC	2	0	0.29(0.09, 0.79)
	UT vs. PA	2	0	0.26(0.08, 0.74)
	UT vs. TFM	1	NA	0.23(0.04, 0.98)
**Outcomes**	**Pairwise meta-analysis**	**No of study**	**Heterogeneity (%)**	**MD 95%CrI**
FEV1	UT vs. AA	10	97.5	−0.33(−0.56, −0.10)
	UT vs. AA_EPE	2	91.7	−0.49(−1.00, −0.02)
	UT vs. AA_TCM	5	71.2	−0.35 (−0.68, −0.03)
	UT vs. AC	6	98.6	−0.43 (−0.73, −0.14)
	UT vs. EAC	1	NA	−0.30 (−1.00, 0.40)
	UT vs. PA	2	0	−0.12 (−0.64, 0.40)
	UT vs. TENS	1	NA	0.05 (−0.72, 0.81)
	UT vs. TFM	1	NA	−0.71 (−1.44, 0.02)
	WA vs. UT	1	NA	0.32 (−0.39, 1.03)
**Outcomes**	**Pairwise meta-analysis**	**No of study**	**Heterogeneity (%)**	**MD 95%CrI**
FVC	UT vs. AA	7	85.2	−0.31 (−0.61, −0.19)
	UT vs. AA_EPE	2	0	−0.36 (−0.91, 0.20)
	UT vs. AA_TCM	6	51.6	−0.39 (−0.72, −0.06)
	UT vs. AC	9	95.7	−0.58 (−0.86, −0.30)
	UT vs. EAC	1	NA	−0.45 (−1.22, 0.33)
	UT vs. PA	1	NA	−0.16 (−0.98, 0.66)
	UT vs. TENS	1	NA	0.02 (−0.87, 0.90)
	WA vs. UT	1	NA	0.33 (−0.44, 1.10)
**Outcomes**	**Pairwise meta-analysis**	**No of study**	**Heterogeneity (%)**	**MD 95%CrI**
FEV1/FVC	UT vs. AA	10	95.5	−5.07 (−7.55, −1.63)
	UT vs. AA_EPE	2	69.6	−5.07 (−9.65, −0.50)
	UT vs. AA_TCM	8	78.5	−5.60 (−8.09, −3.27)
	UT vs. AC	5	95.7	−5.22(−8.28, −2.32)
	UT vs. EAC	2	94.4	−9.38(−14.20, −4.63)
	UT vs. PA	2	8.2	−4.47 (−9.50, 0.50)
	UT vs. TFM	1	NA	−5.93 (−12.56, 0.68)
	WA vs. UT	1	NA	6.20 (−0.30, 12.68)

### Results of consistency modeling

The current study used a random-effects model to compare the difference in DIC between the consistency and inconsistency models was less than 5. The results (Supplementary Table S3) indicate that symptom improvement, FEV1, FVC, and FEV1/FVC have consistency.

### Symptom improvement

**FIGURE 2:**
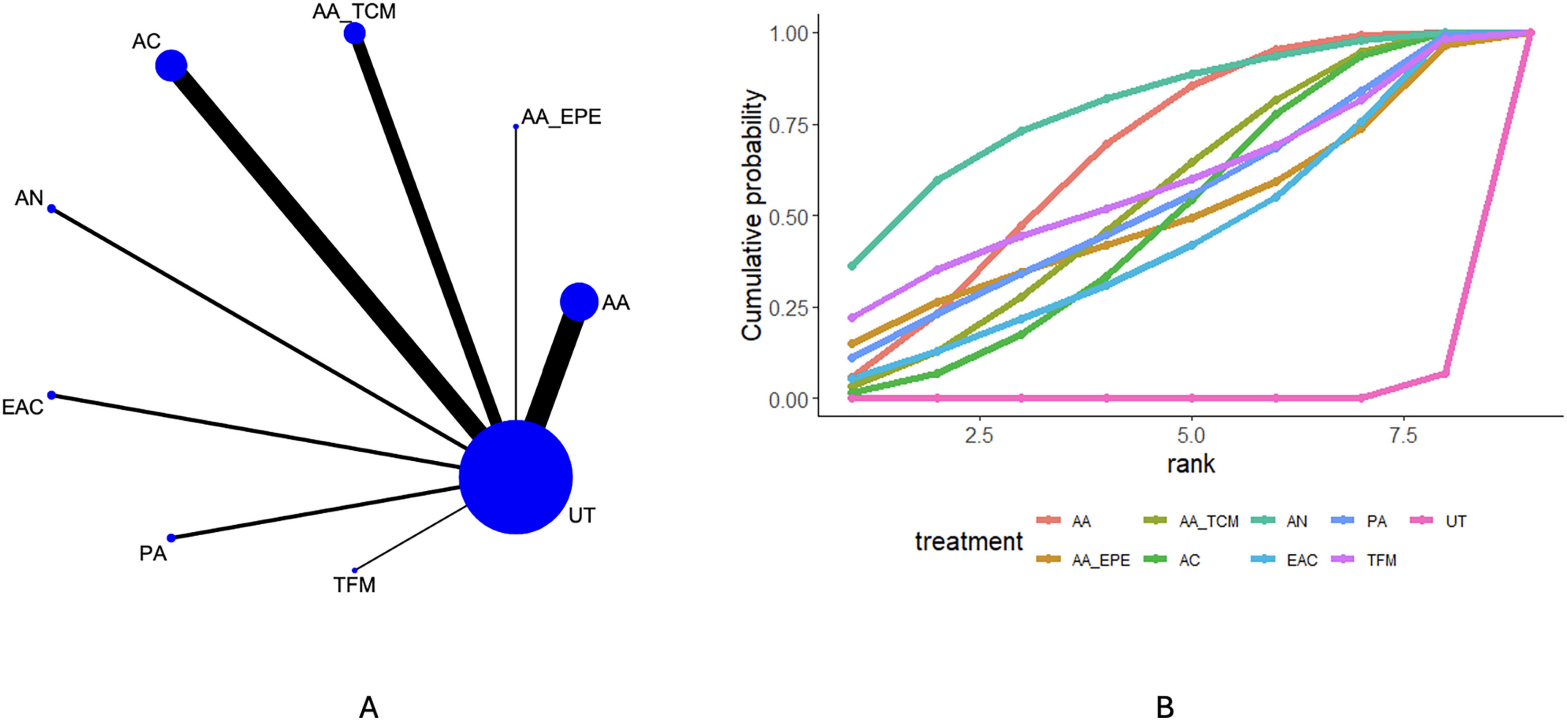
Results of network meta-analysis of efficacy. **(A)** Network plot. **(B)** Cumulative probability ranking plot.

Thirty-four studies reported symptom improvement. The network structure for this outcome is shown in Figure 2A. Compared with usual treatment, AA (OR = 4.97, 95% CrI 3.18–8.24), AA_TCM (OR = 4.24, 95% CrI 2.38–7.82), and AN (OR = 6.84, 95% CrI 2.59–21.54) were associated with higher odds of symptom improvement. No statistically robust differences were observed between most active interventions. Ranking probabilities based on the cumulative ranking curves (Figure 2B and Table 3) suggested that AN, AA, and TFM were more likely to rank among the relatively higher positions for symptom improvement within the network. However, these ranking results reflect relative probabilities under the model and should not be interpreted as definitive evidence of clinical superiority.

**TABLE 3:** SUCRA rank results.

Treatment	E (%)	FEV1 (%)	FVC	FEVFVC
AA	65.73	88.97	31.98	92.11
AA_EPE	49.53	63.24	61.59	44.34
AA_TCM	53.83	49.59	68.74	41.02
AC	48.05	74.78	97.08	14.69
AN	78.88	NR	NR	NR
EAC	42.79	41.66	85.43	90.42
PA	52.60	21.46	28.91	36.17
TENS	NR	6.40	12.63	NR
TFM	57.72	99.56	NR	63.66
UT	0.88	7.08	8.39	0.01
WA	NR	47.26	55.24	67.57

### FEV1

**FIGURE 3:**
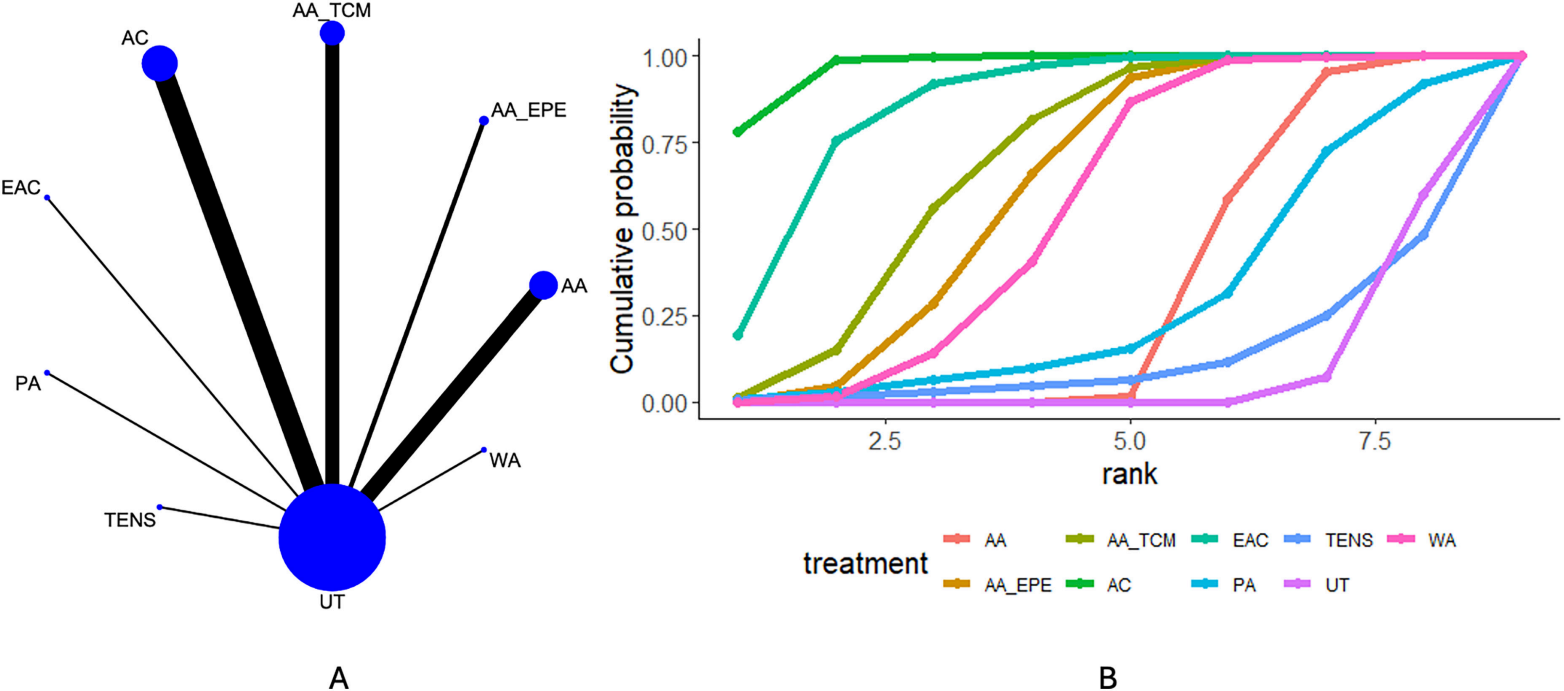
Results of network meta-analysis of FEV_1_. **(A)** Network plot. **(B)** Cumulative probability ranking plot.

Twenty-nine articles reported FEV_1_. The network diagram (Figure 3A) shows direct comparisons among TFM, TENS, PA, EAC, AC, AA_TCM, AA_EPE, AA, WA, and UT. The league table (Supplementary Table S5) indicates that, compared with UT, AA [MD = 0.50, 95% CrI (0.47, 0.54)], AA_EPE [MD = 0.38, 95% CrI (0.30, 0.47)], AA_TCM [MD = 0.33, 95% CrI (0.25, 0.41)], AC [MD = 0.43, 95% CrI (0.37, 0.48)], EAC [MD = 0.30, 95% CrI (0.21, 0.39)], PA [MD = 0.15, 95% CrI (0.01, 0.29)], and TFM [MD = 0.71, 95% CrI (0.50, 0.92)] were associated with higher FEV_1_ values in patients with AECOPD. In addition, AA was associated with higher FEV_1_ values than AA_EPE [MD = 0.12, 95% CrI (0.03, 0.21)], AC than EAC [MD = 0.13, 95% CrI (0.02, 0.24)], AA_TCM than PA [MD = 0.19, 95% CrI (0.03, 0.35)], and AA_EPE than PA [MD = 0.24, 95% CrI (0.07, 0.40)] and TENS [MD = 0.43, 95% CrI (0.10, 0.77)]. Ranking probabilities based on the cumulative ranking curves (Figure 3B and Table 3) suggested that TFM, AA, and AC were more likely to rank relatively higher for FEV_1_ within the network. Nevertheless, SUCRA-based rankings represent the probability of occupying a higher rank rather than the magnitude of treatment effect and therefore should be interpreted cautiously.

### FVC

**FIGURE 4:**
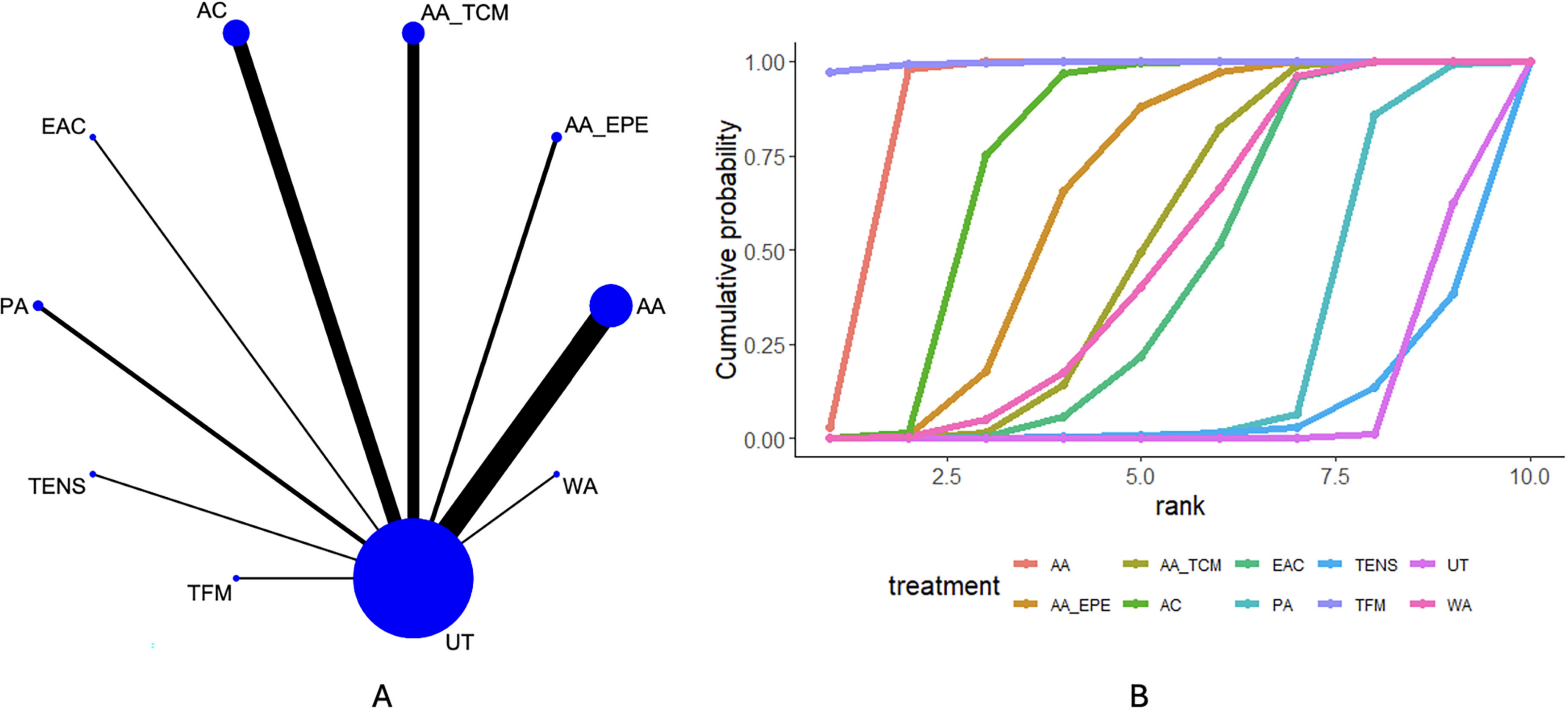
Results of network meta-analysis of FVC. **(A)** Network plot. **(B)** Cumulative probability ranking plot.

Twenty-eight articles reported FVC. The network diagram (Figure 4A) shows direct comparisons among TENS, PA, EAC, AC, AA_TCM, AA_EPE, AA, WA, and UT. The league table (Supplementary Table S6) indicates that, compared with UT, AA [MD = 0.23, 95% CrI (0.18, 0.27)], AA_EPE [MD = 0.35, 95% CrI (0.27, 0.43)], AA_TCM [MD = 0.38, 95% CrI (0.28, 0.48)], AC [MD = 0.51, 95% CrI (0.44, 0.57)], and EAC [MD = 0.45, 95% CrI (0.33, 0.57)] were associated with higher FVC values in patients with AECOPD. In addition, AC was associated with higher FVC values than PA [MD = 0.35, 95% CrI (0.05, 0.64)], TENS [MD = 0.53, 95% CrI (0.10, 0.96)], and WA [MD = 0.18, 95% CrI (0.07, 0.28)]. Ranking probabilities based on the cumulative ranking curves (Figure 4B and Table 3) suggested that AC, EAC, and AA_TCM were more likely to rank among the relatively higher positions for FVC within the network. However, these rankings should be interpreted cautiously because they reflect probabilistic ordering within the model rather than definitive comparative superiority between interventions.

### FEV1/FVC

**FIGURE 5:**
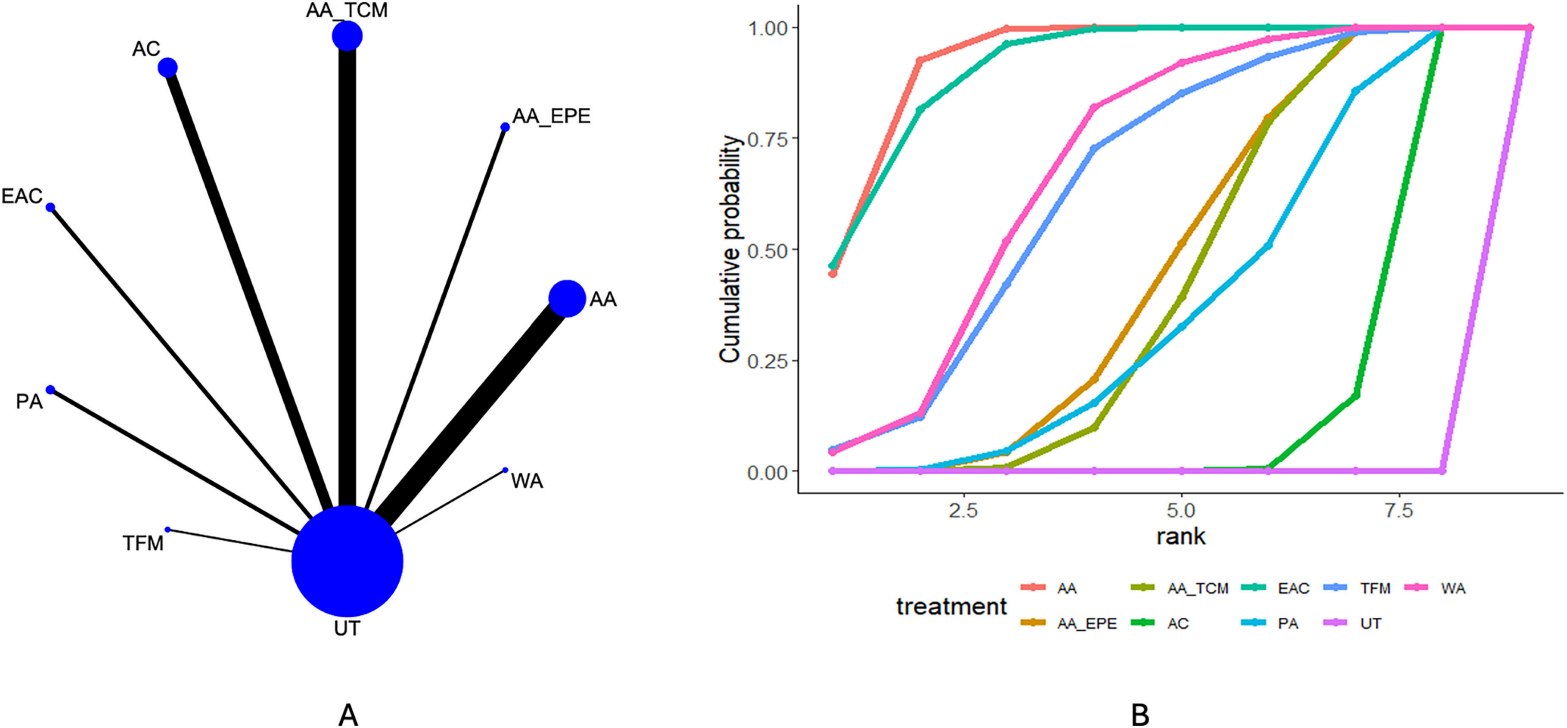
Results of network meta-analysis of FEV_1_/FVC. **(A)** Network plot. **(B)** Cumulative probability ranking plot.

Thirty-one articles reported FEV_1_/FVC. The network diagram (Figure 5A) shows direct comparisons among TFM, PA, EAC, AC, AA_TCM, AA_EPE, AA, WA, and UT. The league table (Supplementary Table S7) indicates that, compared with UT, AA [MD = 7.99, 95% CrI (7.54, 8.43)], AA_EPE [MD = 4.65, 95% CrI (3.04, 6.26)], AA_TCM [MD = 4.48, 95% CrI (3.50, 5.45)], AC [MD = 2.73, 95% CrI (2.09, 3.36)], EAC [MD = 7.96, 95% CrI (6.02, 9.88)], PA [MD = 4.11, 95% CrI (1.61, 6.60)], and TFM [MD = 5.95, 95% CrI (3.24, 8.64)] were associated with higher FEV_1_/FVC values in patients with AECOPD. In addition, AA was associated with higher FEV_1_/FVC values than AA_EPE [MD = 3.34, 95% CrI (1.67, 5.00)], AA_TCM [MD = 3.51, 95% CrI (2.44, 4.58)], AC [MD = 5.26, 95% CrI (4.48, 6.04)], and PA [MD = 3.88, 95% CrI (1.35, 6.42)]. Ranking probabilities based on the cumulative ranking curves (Figure 5B and Table 3) suggested that AA, EAC, and WA were more likely to rank relatively higher for FEV_1_/FVC within the network. However, these rankings should be interpreted cautiously because ranking probabilities do not directly indicate treatment effect magnitude or definitive clinical superiority.

### Publication bias

This study employed funnel plots to detect publication bias. The results (Supplementary Figures S3–S6) indicate symmetrical funnel plots, suggesting a low likelihood of publication bias.

## Discussion

Overall, several acupuncture-related interventions were associated with improvements in symptom-related outcomes and pulmonary function parameters compared with usual treatment. However, differences between interventions should be interpreted cautiously given the uncertainty of comparative estimates and the probabilistic nature of treatment rankings. Therefore, the results support a potential complementary role for these interventions during AECOPD rather than establishing them as standalone therapies.

For symptom improvement, AN showed a relatively higher probability of ranking among the more favorable interventions in the network analysis. Previous study (63) has suggested that AN may help alleviate typical symptoms of AECOPD, such as cough and wheezing, possibly through mechanisms related to the regulation of airway inflammation and bronchial spasm. Although AA and AA_TCM were also associated with improvements in symptom-related outcomes, differences between interventions should be interpreted cautiously given the uncertainty of comparative estimates. From a mechanistic perspective, AN may influence airway responsiveness through modulation of autonomic activity and circulation-related pathways described in traditional Chinese medicine theory (64). However, these findings should be interpreted as exploratory rather than definitive evidence supporting a specific intervention.

Regarding FEV_1_ improvement, TFM showed a relatively higher-ranking probability in the network analysis. TFM is considered a specialized form of acupuncture that promotes qi and blood circulation through its warming effect, thereby enhancing airway ventilation (65). Its potential benefits for FEV_1_ may stem from warming stimulation that promotes airway dilation and pulmonary ventilation. TFM may also enhance pulmonary blood circulation and oxygen supply, which could contribute to improvements in lung function during acute exacerbations (65). However, these interpretations should be considered in the context of the probabilistic nature of treatment rankings.

In this network meta-analysis, treatment rankings were estimated using the surface under the cumulative ranking curve (SUCRA) to provide a probabilistic hierarchy of interventions. However, SUCRA values should not be interpreted as measures of treatment effect size or definitive indicators of clinical superiority. Presenting SUCRA values as highly precise percentages (99.56%) may create a misleading impression of certainty. Instead, SUCRA reflects the probability that an intervention ranks among the more favorable options within the model, conditional on the available evidence. For example, although TFM achieved a high SUCRA ranking for improvement in FEV_1_, the corresponding effect estimate (MD = 0.71, 95% CrI 0.50–0.92 vs. usual treatment) should be interpreted cautiously. The magnitude of effect, overlap of credible intervals across comparisons, and the low certainty of evidence limit confidence in asserting true superiority. Therefore, rankings are best understood as exploratory and hypothesis-generating rather than conclusive evidence of a single “best” intervention.

Regarding FVC, AC showed a relatively higher-ranking probability in the network analysis, followed by EAC and AA_TCM. These findings suggest that several acupuncture-related interventions may be associated with improvements in lung expansion and vital capacity in patients with AECOPD. Potential mechanisms may include modulation of airway inflammation, improvement in airway patency, and enhanced respiratory muscle coordination (66). However, given the overlap of credible intervals across several comparisons, the relative advantages of individual interventions remain uncertain and should be interpreted cautiously (67).

Regarding improvements in the FEV_1_/FVC ratio, AA ranked relatively higher in the network analysis. AA may enhance airway patency and pulmonary gas exchange through stimulation of specific acupoints and transdermal absorption of herbal components (68, 69). During AECOPD, improvement in the FEV_1_/FVC ratio reflects better airway patency and ventilation efficiency, which may contribute to improved respiratory function (70). However, the clinical interpretation of these findings should consider the limitations of the available evidence and the uncertainty inherent in comparative estimates.

To enhance clinical interpretability, we compared pooled effect estimates with established minimal clinically important difference (MCID) thresholds where available. For FEV_1_, an MCID of approximately 0.1 L has been suggested in patients with COPD. In our analysis, the observed mean differences approached or exceeded this threshold in some comparisons, suggesting potential clinical relevance. However, for outcomes where the pooled effect did not clearly surpass MCID thresholds, the clinical impact should be interpreted cautiously despite statistical significance.

Furthermore, although ranking probabilities were estimated in the network meta-analysis, differences between interventions should be interpreted carefully. In several comparisons, overlapping credible intervals were observed, indicating uncertainty regarding the relative advantages of specific interventions. Therefore, rankings reflect probabilistic estimates rather than definitive evidence of a single “best” treatment.

Given the multiple comparisons inherent in network meta-analysis, the possibility of false-positive findings cannot be entirely excluded, and conclusions should be considered hypothesis-generating rather than definitive.

### Strengths and limitations

This study systematically synthesized available randomized evidence on acupuncture-related interventions for AECOPD using a Bayesian network meta-analysis framework. By comparing multiple interventions within a connected network, it provided a broader comparative perspective than conventional pairwise meta-analysis alone.

This study has several limitations. First, its reliance on published literature may introduce publication bias, potentially affecting the generalizability of findings. Second, variations in intervention protocols, treatment frequency, and practitioner experience across studies may account for differences in treatment outcomes, highlighting the absence of standardized treatment protocols. Furthermore, the lack of long-term follow-up data in existing literature prevents assessment of the interventions’ long-term symptom improvement and safety. An important limitation of this network meta-analysis is that several interventions represented combination strategies (acupuncture combined with other TCM therapies or conventional medicine) rather than acupuncture modalities in isolation. Consequently, comparisons such as AA_TCM versus UT evaluate integrated treatment packages instead of the independent contribution of acupuncture. Therefore, the observed differences cannot be solely attributed to acupuncture itself. The conclusions of this study should be interpreted as applying to these specific combination strategies. Future trials designed to isolate the incremental effect of acupuncture are warranted.

## Conclusion

In patients with AECOPD, several acupuncture-related interventions were associated with improvements in symptom-related outcomes and pulmonary function parameters when used in addition to usual treatment. AN, TFM, AC, and AA/EAC showed relatively higher-ranking probabilities for some outcomes, but these rankings do not establish clinical superiority and should be interpreted together with effect estimates and certainty of evidence. Overall, the current evidence supports a possible adjunctive role for acupuncture-related interventions in AECOPD, while the comparative advantages of specific strategies remain uncertain. Further large-scale, rigorously designed, and transparently reported randomized controlled trials are needed to confirm these findings.

## Data Availability

The original contributions presented in the study are included in the article/Supplementary material, further inquiries can be directed to the corresponding authors.
