# Supplementary material for: Efficacy of acupuncture in managing acute exacerbation of chronic obstructive pulmonary disease: a network meta-analysis
Source: Front Med (Lausanne). 2026 Apr 21;13:1786971. doi: 10.3389/fmed.2026.1786971 (PMC13140098; doi:10.3389/fmed.2026.1786971)
Supplement: Supplementary file 1 [file Data_Sheet_1.docx]

Table S1 Search history

(("Acupuncture"[Mesh]) OR ((((((((((((((((((((((((((((((Acupuncture[Title/Abstract]) OR (Pharmacopuncture[Title/Abstract])) OR (Acupuncture Therapy[Title/Abstract])) OR (Acupuncture Treatment[Title/Abstract])) OR (Acupuncture Treatments[Title/Abstract])) OR (Treatment, Acupuncture[Title/Abstract])) OR (Therapy, Acupuncture[Title/Abstract])) OR (Pharmacoacupuncture Treatment[Title/Abstract])) OR (Treatment, Pharmacoacupuncture[Title/Abstract])) OR (Pharmacoacupuncture Therapy[Title/Abstract])) OR (Therapy, Pharmacoacupuncture[Title/Abstract])) OR (Acupotomy[Title/Abstract])) OR (Acupotomies[Title/Abstract])) OR (Acupuncture, Ear[Title/Abstract])) OR (Acupunctures, Ear[Title/Abstract])) OR (Ear Acupunctures[Title/Abstract])) OR (Acupuncture, Auricular[Title/Abstract])) OR (Acupunctures, Auricular[Title/Abstract])) OR (Auricular Acupunctures[Title/Abstract])) OR (Auricular Acupuncture[Title/Abstract])) OR (Ear Acupuncture[Title/Abstract])) OR (Acupuncture Points[Title/Abstract])) OR (Acupuncture Point[Title/Abstract])) OR (Point, Acupuncture[Title/Abstract])) OR (Points, Acupuncture[Title/Abstract])) OR (Acupoints[Title/Abstract])) OR (Acupoint[Title/Abstract])) OR (Electroacupuncture[Title/Abstract])) OR (Moxibustion[Title/Abstract])) OR (Moxabustion[Title/Abstract]))) AND (("Pulmonary Disease, Chronic Obstructive"[Mesh]) OR (((((((((((Pulmonary Disease, Chronic Obstructive[Title/Abstract]) OR (Chronic Obstructive Pulmonary Diseases[Title/Abstract])) OR (COPD[Title/Abstract])) OR (Chronic Obstructive Lung Disease[Title/Abstract])) OR (Chronic Obstructive Pulmonary Disease[Title/Abstract])) OR (COAD[Title/Abstract])) OR (Chronic Obstructive Airway Disease[Title/Abstract])) OR (Airflow Obstruction, Chronic[Title/Abstract])) OR (Airflow Obstructions, Chronic[Title/Abstract])) OR (Chronic Airflow Obstructions[Title/Abstract])) OR (Chronic Airflow Obstruction[Title/Abstract])))

Table S2 grade results

| Outcomes | Risk of Bias | Inconsistency | Indirectness | Imprecision | Publication Bias | Grade results |
| --- | --- | --- | --- | --- | --- | --- |
| efficacy | Serious (-1)  Limitations in blinding and allocation concealment in several trials | Not serious (0) | Not serious (0) | Not serious (0) | Undetected (0) | Moderate |
| FEV1 | Serious (-1)  Limitations in blinding and allocation concealment in several trials | Serious (-1)  Heterogeneity across studies | Not serious (0) | Not serious (0) | Undetected (0) | Low |
| FVC | Serious (-1)  Limitations in blinding and allocation concealment in several trials | Serious (-1)  Heterogeneity across studies | Not serious (0) | Not serious (0) | Undetected (0) | Low |
| FEV1/FVC | Serious (-1)  Limitations in blinding and allocation concealment in several trials | Serious (-1)  Heterogeneity across studies | Not serious (0) | Not serious (0) | Undetected (0) | Low |

Table S3 Results of consistency modeling

| Outcomes | Consistency test | Inconsistency test | I^2^(%) |
| --- | --- | --- | --- |
| Efficacy | 104.94 | 104.77 | 0 |
| FEV1 | 739.46 | 739.43 | 92 |
| FVC | 290.62 | 290.50 | 78 |
| FEVFVC | 234.30 | 234.32 | 69 |

table S4 efficacy League table

| OR (95%Crl) | | | | | | | | |
| --- | --- | --- | --- | --- | --- | --- | --- | --- |
| AA |  |  |  |  |  |  |  |  |
| 1.33 (0.23, 5.98) | AA_EPE |  |  |  |  |  |  |  |
| 1.18 (0.55, 2.5) | 0.89 (0.19, 5.28) | AA_TCM |  |  |  |  |  |  |
| 1.27 (0.65, 2.5) | 0.96 (0.21, 5.42) | 1.08 (0.5, 2.28) | AC |  |  |  |  |  |
| 0.74 (0.21, 2.16) | 0.55 (0.09, 3.97) | 0.62 (0.17, 1.94) | 0.58 (0.17, 1.75) | AN |  |  |  |  |
| 1.46 (0.44, 4.59) | 1.11 (0.18, 7.91) | 1.24 (0.35, 4.31) | 1.15 (0.34, 3.68) | 2 (0.46, 9.39) | EAC |  |  |  |
| 1.22 (0.34, 4.08) | 0.92 (0.14, 7.15) | 1.04 (0.28, 3.66) | 0.96 (0.27, 3.35) | 1.65 (0.36, 8.35) | 0.83 (0.17, 4.09) | PA |  |  |
| 1.09 (0.16, 5) | 0.83 (0.08, 7.56) | 0.93 (0.14, 4.44) | 0.86 (0.13, 4.09) | 1.48 (0.18, 9.8) | 0.74 (0.1, 4.62) | 0.88 (0.12, 5.82) | TFM |  |
| 4.97 (3.18, 8.24)* | 3.74 (0.89, 20.12) | 4.24 (2.38, 7.82)* | 3.93 (2.48, 6.5)* | 6.84 (2.59, 21.54)* | 3.42 (1.21, 10.78)* | 4.1 (1.33, 13.55)* | 4.55 (1.07, 28.56)* | UT |

Means P<0.05

table S5 FEV1 League table

| MD (95%Crl) | | | | | | | | | |
| --- | --- | --- | --- | --- | --- | --- | --- | --- | --- |
| AA |  |  |  |  |  |  |  |  |  |
| 0.12 (0.03,0.21)* | AA_EPE |  |  |  |  |  |  |  |  |
| 0.17 (0.08, 0.26) * | 0.05 (-0.07, 0.17) | AA_TCM |  |  |  |  |  |  |  |
| 0.07 (0.01, 0.14) * | -0.05 (-0.15, 0.06) | -0.1 (-0.19, 0) | AC |  |  |  |  |  |  |
| 0.2 (0.1, 0.3) * | 0.08 (-0.04, 0.21) | 0.03 (-0.09, 0.16) | 0.13 (0.02, 0.24) * | EAC |  |  |  |  |  |
| 0.36 (0.21, 0.5) * | 0.24 (0.07, 0.4) * | 0.19 (0.03, 0.35) * | 0.28 (0.13, 0.43) * | 0.15 (-0.01, 0.32) | PA |  |  |  |  |
| 0.55 (0.23, 0.88) * | 0.43 (0.1, 0.77) * | 0.38 (0.05, 0.71) * | 0.48 (0.15, 0.81) * | 0.35 (0.01, 0.69) * | 0.2 (-0.15, 0.55) | TENS |  |  |  |
| -0.21 (-0.42, 0.01) | -0.33 (-0.55, -0.1) * | -0.38 (-0.6, -0.15) * | -0.28 (-0.5, -0.06) * | -0.41 (-0.64, -0.18) * | -0.56 (-0.82, -0.31) * | -0.76 (-1.15, -0.37) * | TFM |  |  |
| 0.5 (0.47, 0.54) * | 0.38 (0.3, 0.47) * | 0.33 (0.25, 0.41) * | 0.43 (0.37, 0.48) * | 0.3 (0.21, 0.39) * | 0.15 (0.01, 0.29) * | -0.05 (-0.37, 0.27) | 0.71 (0.5, 0.92) * | UT |  |
| 0.18 (0.05, 0.31) * | 0.06 (-0.09, 0.22) | 0.01 (-0.14, 0.16) | 0.11 (-0.03, 0.25) | -0.02 (-0.18, 0.14) | -0.17 (-0.36, 0.01) | -0.37 (-0.72, -0.02) * | 0.39 (0.14, 0.64) * | -0.32 (-0.45, -0.19) * | WA |

Means P<0.05

table S6 FVC League table

| MD (95%Crl) | | | | | | | |  |
| --- | --- | --- | --- | --- | --- | --- | --- | --- |
| AA |  |  |  |  |  |  |  |  |
| -0.13 (-0.22, -0.04)* | AA_EPE |  |  |  |  |  |  |  |
| -0.15 (-0.26, -0.04) * | -0.03 (-0.15, 0.1) | AA_TCM |  |  |  |  |  |  |
| -0.28 (-0.36, -0.2) * | -0.16 (-0.26, -0.05) * | -0.13 (-0.25, -0.01) * | AC |  |  |  |  |  |
| -0.22 (-0.35, -0.1) * | -0.1 (-0.24, 0.04) | -0.07 (-0.22, 0.08) | 0.06 (-0.08, 0.19) | EAC |  |  |  |  |
| 0.06 (-0.23, 0.36) | 0.19 (-0.11, 0.49) | 0.22 (-0.09, 0.52) | 0.35 (0.05, 0.64) * | 0.29 (-0.02, 0.6) | PA |  |  |  |
| 0.25 (-0.18, 0.68) | 0.37 (-0.06, 0.81) | 0.4 (-0.04, 0.83) | 0.53 (0.1, 0.96) * | 0.47 (0.03, 0.91) * | 0.18 (-0.34, 0.69) | TENS |  |  |
| 0.23 (0.18, 0.27) * | 0.35 (0.27, 0.43) * | 0.38 (0.28, 0.48) * | 0.51 (0.44, 0.57) * | 0.45 (0.33, 0.57) * | 0.16 (-0.13, 0.45) | -0.02 (-0.45, 0.41) | UT |  |
| -0.1 (-0.2, -0.01) * | 0.02 (-0.09, 0.14) | 0.05 (-0.08, 0.17) | 0.18 (0.07, 0.28) * | 0.12 (-0.02, 0.26) | -0.17 (-0.47, 0.13) | -0.35 (-0.78, 0.08) | -0.33 (-0.41, -0.25) * | WA |

Means P<0.05

table S7 FEVFVC League table

| MD (95%Crl) | | | | | | | | |
| --- | --- | --- | --- | --- | --- | --- | --- | --- |
| AA |  |  |  |  |  |  |  |  |
| 3.34 (1.67, 5)* | AA_EPE |  |  |  |  |  |  |  |
| 3.51 (2.44, 4.58) * | 0.17 (-1.7, 2.05) | AA_TCM |  |  |  |  |  |  |
| 5.26 (4.48, 6.04) * | 1.92 (0.2, 3.65) * | 1.75 (0.59, 2.91) * | AC |  |  |  |  |  |
| 0.03 (-1.95, 2.01) | -3.31 (-5.81, -0.8) * | -3.48 (-5.63, -1.32) * | -5.23 (-7.26, -3.2) * | EAC |  |  |  |  |
| 3.88 (1.35, 6.42) * | 0.54 (-2.41, 3.5) | 0.37 (-2.32, 3.04) | -1.38 (-3.95, 1.2) | 3.85 (0.7, 6.99) * | PA |  |  |  |
| 2.04 (-0.7, 4.78) | -1.3 (-4.44, 1.84) | -1.47 (-4.33, 1.39) | -3.22 (-6, -0.44) * | 2.01 (-1.29, 5.34) | -1.84 (-5.49, 1.82) | TFM |  |  |
| 7.99 (7.54, 8.43) * | 4.65 (3.04, 6.26) * | 4.48 (3.5, 5.45) * | 2.73 (2.09, 3.36) * | 7.96 (6.02, 9.88) * | 4.11 (1.61, 6.6) * | 5.95 (3.24, 8.64) * | UT |  |
| 1.78 (-0.56, 4.14) | -1.56 (-4.36, 1.26) | -1.72 (-4.23, 0.79) | -3.47 (-5.86, -1.08) * | 1.75 (-1.25, 4.76) | -2.09 (-5.48, 1.31) | -0.25 (-3.81, 3.3) | -6.2 (-8.5, -3.89) * | WA |

Means P<0.05


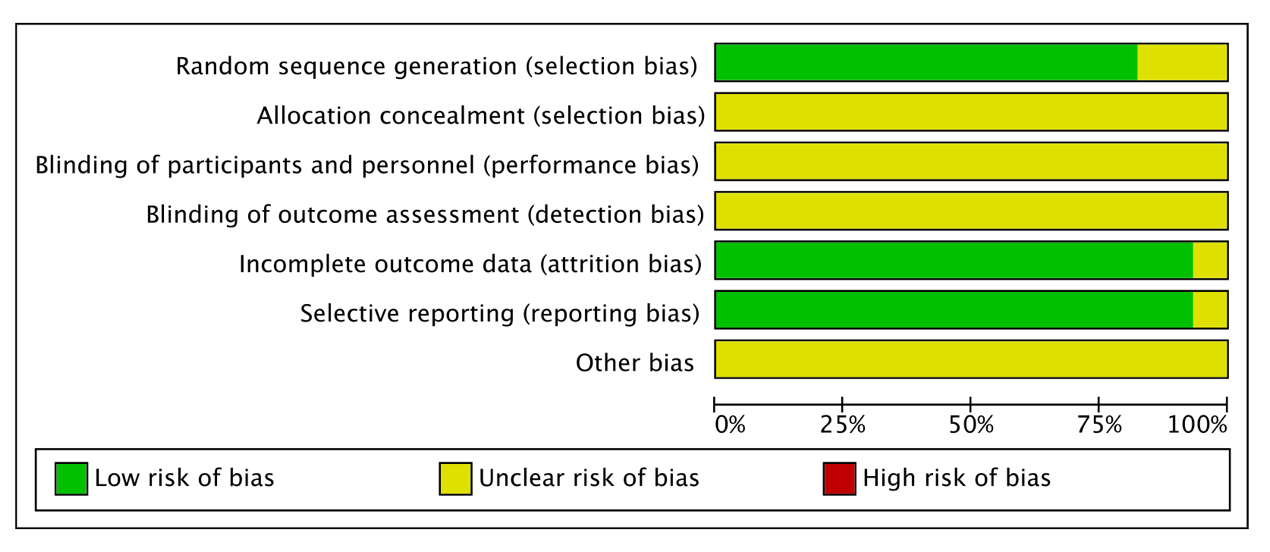


Figure S1 risk bias of graph


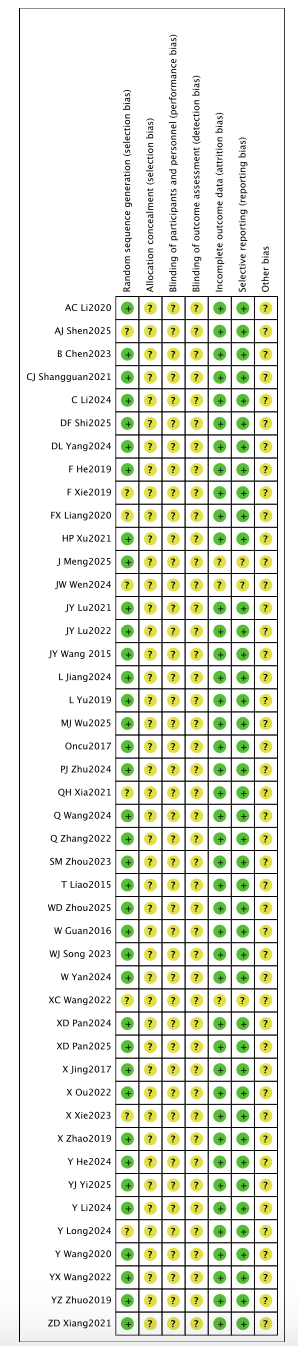


Figure S2 risk bias of summary


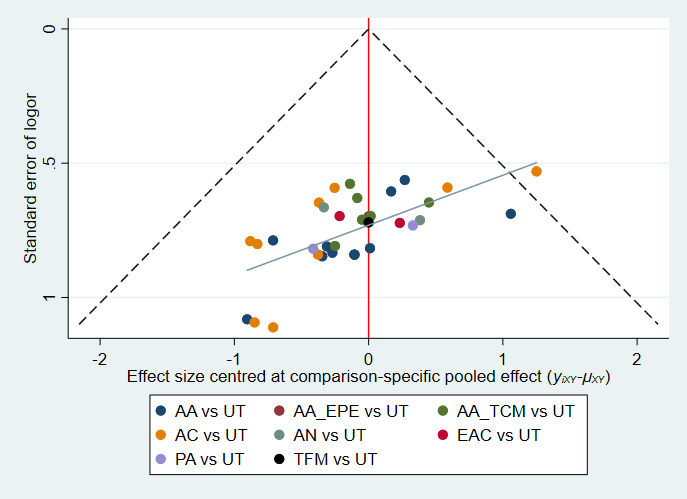


Figure S3 Funnel plot of meta-analysis of efficacy


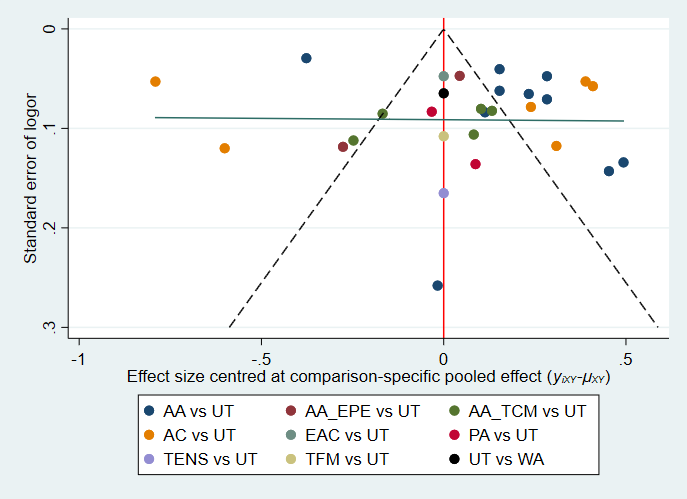


Figure S4 Funnel plot of meta-analysis of FEV1


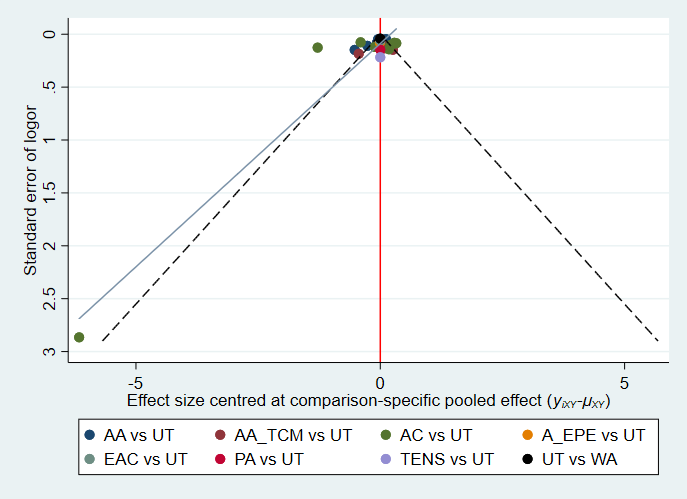


Figure S5 Funnel plot of meta-analysis of FVC


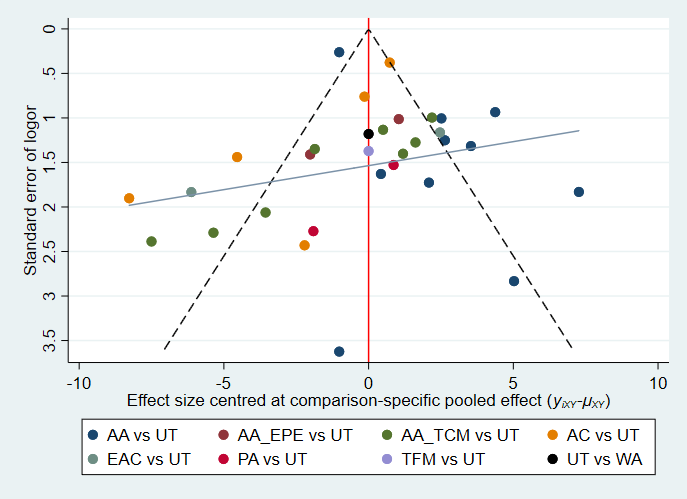


Figure S6 Funnel plot of meta-analysis of FEV1/FVC
